# The role of animals as a source of antimicrobial resistant nontyphoidal *Salmonella* causing invasive and non-invasive human disease in Vietnam

**DOI:** 10.1016/j.meegid.2020.104534

**Published:** 2020-11

**Authors:** Andrea Parisi, Tu Le Thi Phuong, Alison E. Mather, Thibaut Jombart, Ha Thanh Tuyen, Nguyen Phu Huong Lan, Nguyen Hoang Thu Trang, Juan Carrique-Mas, James I. Campbell, Nguyen Vinh Trung, Kathryn Glass, Martyn D. Kirk, Stephen Baker

**Affiliations:** a Research School of Population Health, Australian National University, Australia; b The Hospital for Tropical Diseases, Wellcome Trust Major Overseas Programme, Oxford University Clinical Research Unit, Ho Chi Minh City, Viet Nam; c Quadram Institute Bioscience, Norwich, United Kingdom; d The London School of Hygiene and Tropical Medicine, London, United Kingdom; e The Hospital for Tropical Diseases, Ho Chi Minh City, Viet Nam; f Centre for Tropical Medicine and Global Health, Nuffield Department of Clinical Medicine, Oxford University, Oxford, United Kingdom; g Cambridge Institute of Therapeutic Immunology & Infectious Disease (CITIID), Cambridge Biomedical Campus, University of Cambridge, Cambridge, United Kingdom

**Keywords:** Nontyphoidal *Salmonella*, Bacteremia, Diarrhea, Antimicrobial resistance, Zoonosis

## Abstract

**Background:**

Nontyphoidal *Salmonella* (NTS) are associated with both diarrhea and bacteremia. Antimicrobial resistance (AMR) is common in NTS in low-middle income countries, but the major source(s) of AMR NTS in humans are not known. Here, we aimed to assess the role of animals as a source of AMR in human NTS infections in Vietnam. We retrospectively combined and analyzed 672 NTS human and animal isolates from four studies in southern Vietnam and compared serovars, sequence types (ST), and AMR profiles. We generated a population structure of circulating organisms and aimed to attribute sources of AMR in NTS causing invasive and noninvasive disease in humans using Bayesian multinomial mixture models.

**Results:**

Among 672 NTS isolates, 148 (22%) originated from human blood, 211 (31%) from human stool, and 313 (47%) from animal stool. The distribution of serovars, STs, and AMR profiles differed among sources; serovars Enteritidis, Typhimurium, and Weltevreden were the most common in human blood, human stool, and animals, respectively. We identified an association between the source of NTS and AMR profile; the majority of AMR isolates were isolated from human blood (*p* < 0.001). Modelling by ST-AMR profile found chickens and pigs were likely the major sources of AMR NTS in human blood and stool, respectively; but unsampled sources were found to be a major contributor.

**Conclusions:**

Antimicrobial use in food animals is hypothesized to play role in the emergence of AMR in human pathogens. Our cross-sectional population-based approach suggests a significant overlap between AMR in NTS in animals and humans, but animal NTS does explain the full extent of AMR in human NTS infections in Vietnam.

## Highlights

• The major source(s) of antimicrobial resistance (AMR) in nontyphoidal *Salmonella* (NTS) are unknown.
• We analyzed 672 NTS isolates, 148 (41%) from human blood, 211 (59%) from human stool, and 136 (43%) from animal stool.
• Enteritidis, Typhimurium, and Weltevreden were the common serovars in human blood, human stool, and animals, respectively.
• The majority of AMR isolates were associated with human blood.
• NTS from animals does explain the full extent of AMR in human NTS infections.

## 1 Background

*Salmonella* is a genus of Gram-negative bacteria that infect a broad range of host species. *Salmonella enterica* is divided into six subspecies but the majority of organisms that cause infections in mammals belong to subspecies I (Brenner et al., 2000; Su and Chiu, 2007). A small number of *Salmonella* serovars are human restricted and cause a systemic “typhoidal” disease. However, the majority of *Salmonella enterica* have animal reservoirs and are termed as “nontyphoidal” (NTS). Human NTS infections are generally considered to arise through the food chain, but infection can also occur via contact with infected animals, through person-to-person transmission, and contaminated water (World Health Organization, 2018).

Some individuals, including infants and the immunocompromised are at higher risk of invasive *Salmonella* infection with an NTS serovar (iNTS) (Crump et al., 2015). In sub-Saharan Africa, iNTS has become a leading cause of bloodstream infections with specific sequence types (STs) emerging with multi-drug resistant (MDR) phenotypes (Kingsley et al., 2009). As a consequence of the increasing trend of antimicrobial resistance (AMR) in NTS (Lee et al., 2009; Stevenson et al., 2007), *Salmonella* are on the WHO global priority pathogen list for the development of new antimicrobials (World Health Organization, 2017).

Diarrhea is a common disease among children in Vietnam and 4–15% of all pediatric diarrheal cases are associated with NTS (Thompson et al., 2015). Additionally, Vietnam has a high prevalence of AMR bacteria, which may be attributed to antimicrobial use (AMU) in agriculture and human health. The need to control AMU has been recognized by political and public health bodies in Vietnam (Food and Agriculture Organization of the United Nations, 2017; Ministry of Health, 2017). Although AMR in NTS infections represent an important public health issue in Vietnam, the role of animals as a potential source for specific NTS serovars and AMR profiles associated with human infection has not been investigated. Here, we investigated the population causing human NTS infections in Vietnam and aimed to identify potential sources of AMR in NTS isolated from humans.

## 2 Methods

### 2.1 Sampling

We combined data from 703 *Salmonella* from four studies, i) The Vietnam Initiative on Zoonotic Infections (VIZIONs); a community-based study of zoonotic disease (Rabaa et al., 2015), ii) The bacterial etiology and antimicrobial susceptibility profile of bloodstream infections in Ho Chi Minh City (HCMC), Vietnam, iii) The etiology of diarrhea, hepatitis, respiratory infections, and central nervous system infections; and iv) The Vietnam Bacterial Resistance Study (VIBRE) (Table S1). NTS organisms from the blood of febrile individuals were isolated in HCMC from January 2010 to December 2013 (Phu Huong Lan et al., 2016). NTS organisms from human stools were isolated from children attending three different hospitals in HCMC with acute diarrhea and from non-diarrheal controls admitted between May 2009 and April 2010 (Thompson et al., 2015). Additional NTS organisms from human stools were cultured from asymptomatic poultry farmers and individuals involved in poultry farming from two rural districts and the capital of Tien Giang Province in the Mekong Delta from March 2012 to April 2013 (Trung et al., 2017). Animal NTS isolates were collected from the same 93 poultry farms as human isolates in Tien Giang Province from March 2012 to April 2013 (Trung et al., 2017), and from 341 pig and poultry farms in Dong Thap from February to May 2012 (Nhung et al., 2015; Tu et al., 2015). NTS from animals were isolated on private farms from fecal specimens.

### 2.2 MLST and antimicrobial susceptibility testing

The methods for bacterial culture, identification, Multi Locus Sequence Typing (MLST), and antimicrobial susceptibility testing have been described previously (Thompson et al., 2015; Phu Huong Lan et al., 2016; Trung et al., 2017; Nhung et al., 2015; Tu et al., 2015). MLST was performed by PCR amplification and DNA sequence analysis of seven housekeeping genes (*aroC, dnaN*, *hemD*, *hisD*, *purE*, *sucA*, *thrA*) to generate specific sequence types (STs) and to perform molecular serotyping (Achtman et al., 2012). A minimum spanning tree was created using the allelic profiles of human blood/stool and animal NTS isolates using BioNumerics 7.2 (Applied Maths, Kortrijk, Belgium).

Antimicrobial susceptibility testing was performed using the disk diffusion method against: ampicillin (AMP), amikacin (AK), ceftazidime (CAZ), ceftriaxone (CTX), chloramphenicol (CHL), ciprofloxacin (CIP), gentamicin (GEN), nalidixic acid (NAL), ofloxacin (OFX), and trimethoprim-sulfamethoxazole (SXT). Zone sizes were interpreted according to the 2014 Clinical and Laboratory Standards Institute (CLSI) guidelines (Clinical and Laboratory Standards Institute (CLSI), 2020). MDR was defined as non-susceptibility to ≥1 agents in ≥3 antimicrobial categories (Magiorakos et al., 2012); intermediate isolates were grouped with the resistant isolates.

### 2.3 Data analysis

To maximize the number of organisms available for analysis, isolates with an incomplete antimicrobial susceptibility profile were excluded if they represented ≤10% of all isolates; otherwise, tested antimicrobials accounting for missing susceptibility data in >10% of isolates were excluded from the analysis. Chi square tests were used to test for heterogeneity and trends in the proportion of STs, AMR profiles, and ST-AMR profiles between human blood/stool and animal isolates. *t*-tests were used to compare the means of antimicrobial agents to which isolates from different sources were resistant. Results with *p* ≤ 0.05 were considered significant. The distribution of STs and AMR profiles by the specific source of isolate was graphically presented using ggplots.

To assess the role of serovar and corresponding ST on the manifestation of human NTS infection, we calculated an index of invasiveness, which was the proportion of invasive (blood) isolates to the total number of human isolates (blood+stool) recovered for each serovar/ST. Due to low prevalence of certain serovars, an invasiveness index was calculated only for the serovars comprising ≥10 NTS isolates.

We estimated potential sources of human NTS using Bayesian multinomial mixture modelling fully described in the Supplementary Information Appendix 1. We used two different models for origins of invasive NTS (human blood isolates) and non-invasive NTS (human stool isolates) contrasting ST, AMR, and ST-AMR profiles. In the first model we assumed that all relevant sources have been sampled. In the second model we also allowed for an “unsampled source”. We estimated mixture coefficients α (presented in %) with an associated 95% credibility interval (CrI). Model fit and convergence was visually assessed using the trace of log-posterior values and of mixture coefficients. All analyses were performed using R version 3.5.1.

## 3 Results

### 3.1 Sampling

Of 703 NTS isolates, 31 isolates were excluded based on the incomplete antimicrobial susceptibility profile and other criteria specified above. 672 NTS isolates were available for analysis, 359/672 (53%) originating from humans and 313/672 (47%) from animals. Among the NTS isolates from humans, 148/359 (41%) originated from blood and 211/359 (59%) from stool. In the animal isolates, 136/313 (43%) originated from chickens, 75/313 (24%) from ducks, 65/313 (21%) from pigs, and 37/313 (12%) from rodents.

### 3.2 Serovar and sequence type distribution

**Fig. 1:**
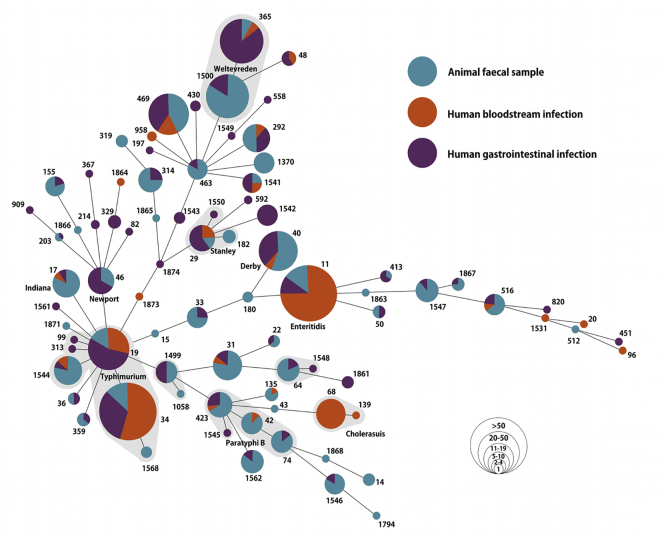
The population structure of NTS isolated from humans and animals in Vietnam. Minimum spanning tree of the 672 different NTS isolates subjected to MLST. The sequence type (ST) of each allelic profile is labelled as are the major inferred serovars. Branch lengths are associated with the number of allelic variations between the STs and clonal complexes are shaded in grey. The source of each isolate (and contribution to each ST) is colour coded and the the size of each ST circle corresponds to the number of isolates per ST profile (scale shown).

We identified 82 unique STs, comprised of 45 different NTS serovars (Tables S2–4). Segregation of the various STs into their origin (animal, human blood, and human stool) depicted a complex structure with some STs overlapping in origin and others being more restricted (Fig. 1). The most common serovars isolated from human blood were Enteritidis (ST11) and Typhimurium (ST34, 19, and 1544) accounting for 62/148 (42%) and 44/148 (30%) of human blood isolates, respectively (Table S2). From the human stool samples, 46/211 (22%) of isolates were *S.* Typhimurium (ST19, 34, 36, 99, 313, and 1544) and 41/211 (20%) were *S.* Weltevreden (ST365 and 1500) (Table S3). Weltevreden (ST1500 and 365) and Typhimurium (ST1544, 19, 34, and 36) were also the most commonly isolated serovars in animals, accounting for 43/313 (14%) and 32/313 (10%) of animal isolates, respectively (Table S4).

We next compared the distribution of NTS STs between humans and animals. From 461 NTS isolates from human blood and animals, we identified 60 different STs; 9 STs were associated with human blood only, 51 STs were associated with animals only, and 16 STs were shared between the human blood and animal isolates. From 524 NTS isolates from human stools and animals, we identified 74 STs; 23 STs were unique to human stool, 18 STs were unique to animals, and 33 STs were shared by both (Fig. S1).

Both serovar and ST were significantly associated with invasiveness of human NTS isolates (*p* < 0.001; Chi-squared test). Invasiveness index for serovars with ≥10 isolates was highest for serovars Choleraesuis (100%), Enteritidis (91%), and Typhimurium (49%). Among *S*. Typhimurium isolates, the most invasive STs with ≥10 isolates per category were ST34 and 19 with invasiveness indices of 58% and 40%, respectively (Table 1).

**Table 1:** Invasiveness index of human NTS serovars and sequence types in Vietnam.

Serovar^a^	Isolates (n)	Invasive isolates (Invasiveness index, %)
Typhimurium	90	44 (48.89)
ST 34	48	28 (58.33)
ST 19	35	14 (40)
ST 1544	3	3 (66.67)
ST 36	2	0 (0)
ST 99	1	0 (0)
ST 313	1	0 (0)
Enteritidis	68	62 (91.18)
ST 11	68	62 (91.18)
Weltevreden	42	1 (2.38)
ST 365	34	1 (2.94)
ST 1500	8	1 (0)
Choleraesuis	15	15 (100)
ST 68	14	14 (100)
ST 139	1	1 (100)
Newport	12	1 (8.33)
ST 46	9	0 (0)
ST 31	3	1 (33.33)
Stanley	12	3 (25)
ST 29	11	3 (27.27)
ST 1550	1	0 (0)
Derby	11	1 (9.09)
ST 40	11	1 (9.09)
Rissen	11	3 (27.27)
ST 469	11	3 (27.07)
N/A		
ST 1542	10	0 (0)

a Analyses were performed only for the serovars with 10≥ isolates per category.

**Fig. 2:**
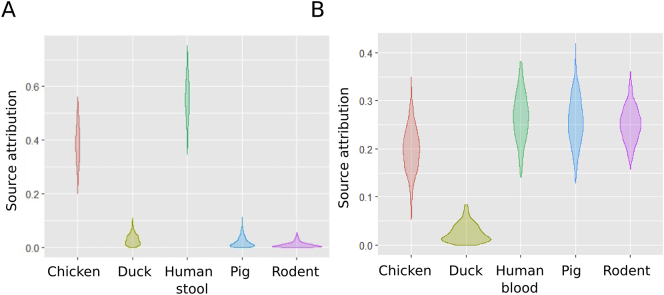
Source attribution of human NTS isolates in Vietnam by sequence type. Violin plots showing the results of the source attribution model for NTS infections in human blood (A) and human stool (B). Each plot represents the mixture coefficient (α), which is an estimated proportion of NTS cases attributed to each source according to sequence type using Bayesian multinomial mixture modelling with sampled sources. The centre of each violin represents the median, the length represents credibility interval and the shape displays frequencies of values. Each of the sampled sources is labelled on the x axis and the proportional contribution (source attribution) is labelled on the y axis.

We aimed to identify the potential origin of NTS in human blood by modelling the ST data. Our model found that 56% (95% CrI 41.3–67.6) of blood infections could be attributed to human non-invasive NTS isolates; chickens were the second most important contributor (39%; CrI 26.4–51.0) (Fig. 2). The results of this model were comparable when an unsampled source was incorporated (Fig. S2). We performed the same analysis on the NTS isolates from human stool. Humans with invasive disease, pigs, and rodents, were all significant contributors, with overlapping ST profiles ranging from 27% to 25% (Fig. 2). When an unsampled source was included in the model, this was the main contributor, accounting for approximately 55% of NTS in human stool (Fig. S2).

### 3.3 Antimicrobial susceptibility

There was a significant association between source of NTS isolates and antimicrobial susceptibility (*p* < 0.05, Chi-squared test). Exceptions were ceftriaxone (*p* = 0.12, Chi-squared test) and ceftazidime (*p* = 0.23; Chi-squared test) where resistance was <4% in each of the sources. The highest proportion of antimicrobial resistant NTS isolates was in the organisms from human blood (*p* < 0.001; Chi-squared test). Blood isolates were resistant to a mean of 2.5/8 antimicrobials (SD = 2.1), human stool isolates to a mean of 1.2/8 antimicrobials (SD = 1.7), and animal isolates to a mean of 1/8 antimicrobials (SD = 1.5). Consequently, NTS isolates from human blood were significantly more resistant than human stool and animal isolates (*p* < 0.001; *t*-test). Ciprofloxacin resistance was notably high in human blood isolates (76/148; 51%) in comparison to human stool (17/211; 8%) and animal isolates (61/313; 19%) (*p* < 0.001; Chi-squared test). Of the 148 isolates from human blood, 61/148 (41%) were MDR. MDR phenotypes were less common in human stool isolates (50/211; 24%) and animal isolates (47/313; 15%) than human blood isolates (*p* < 0.001; Chi-squared test) (Table 2, Fig. S3).

**Table 2:** The antimicrobial susceptibility profiles of NTS isolates in Vietnam.

Antimicrobial susceptibility profile	Human blood isolates (*n*, %)	Human stool isolates (*n*, %)	Animal isolates (*n*, %)
Total tested	148 (100)	211 (100)	313 (100)
Fully susceptible	28 (18.92)	111 (52.61)	167 (53.35)
R-Ampicillin	98 (66.22)	71 (33.65)	72 (23)
R-Amikacin	21 (14.19)	21 (9.95)	21 (6.71)
R-Ceftazidime	2 (1.35)	7 (3.32)	3 (0.96)
R-Ceftriaxone	4 (2.7)	6 (2.84)	3 (0.96)
R-Chloramphenicol	59 (39.86)	51 (24.17)	71 (22.68)
R-Ciprofloxacin	76 (51.35)	17 (8.06)	61 (19.49)
R-Gentamycin	46 (31.08)	27 (12.8)	18 (5.75)
R-Trimethoprim-sulfamethoxazole	58 (39.19)	53 (25.12)	72 (23)
R-Clinically important agent^a^	120 (81.08)	94 (44.55)	136 (43.45)
MDR^b^	61 (41.22)	50 (23.7)	47 (15.02)

a Resistant to ampicillin, ceftriaxone, ciprofloxacin, gentamicin, and/or trimethoprim-sulfamethoxazole.

b Resistant to ≥1 agent in ≥3 antimicrobial categories including penicillins (ampicillin), aminoglycosides (gentamicin, amikacin), sulphonamides (trimethoprim-sulfamethoxazole), quinolones (ciprofloxacin), cephalosporins (ceftriaxone, ceftazidime), and phenicols (chloramphenicol).

**Fig. 3:**
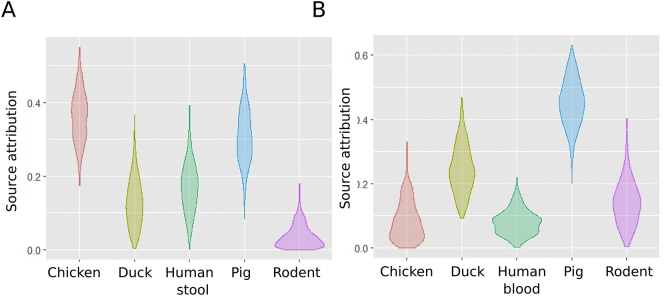
Source attribution of human NTS isolates in Vietnam by AMR profile. Violin plots showing the results of the source attribution model for NTS infections in human blood (A) and human stool (B). Each plot represents the mixture coefficient (α), which is an estimated proportion of NTS cases attributed to each source according to AMR profile using Bayesian multinomial mixture modelling with sampled sources. The centre of each violin represents the median, the length represents credibility interval and the shape displays frequencies of values. Each of the sampled sources is labelled on the x axis and the proportional contribution (source attribution) is labelled on the y axis.

After combining data from all NTS isolates, we identified 43 different antimicrobial susceptibility profiles. Comparing NTS isolates from human blood and animals, 19 profiles were unique to humans, 16 were unique to animals, and 18 were shared. Comparing NTS isolates from human stool and animals, 19 antimicrobial susceptibility profiles were shared by both species, 11 were found in humans only, and 15 in animals only (Fig. S4). (Tables S5–7). Modelling the potential source of AMR in NTS from human blood and stool found that chickens and pigs were the main contributors, accounting for 37% (95% CrI 23.9–48.5) and 45% (95% CrI 30.5–58.7) of NTS cases, respectively (Fig. 3). When allowing for an unsampled source, the most likely origin of AMR in NTS from human blood remained pigs (Fig. S5).

### 3.4 Sequence type-antimicrobial susceptibility profile distribution

**Fig. 4:**
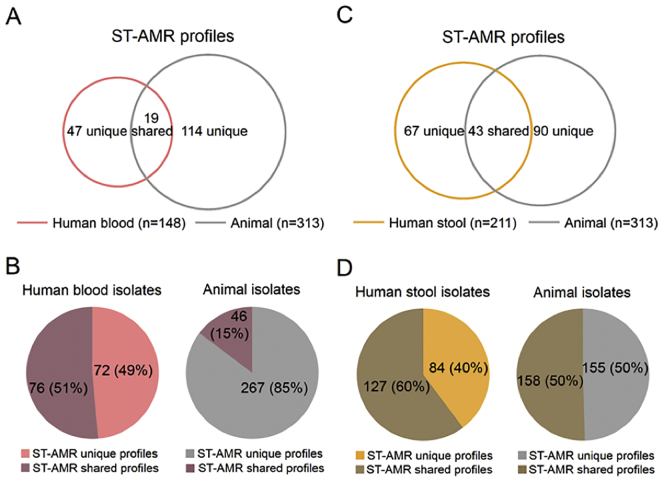
The number and proportion of unique and shared ST-AMR profiles among human and animal NTS isolates in Vietnam. Venn diagrams showing the number of unique and shared ST-AMR profiles between organisms isolated from human blood and animals (A) and human stools and animals (C). Pie charts showing the unique and shared ST-AMR profiles between organisms isolated from human blood and animals (B) and human stools and animals (D).

**Fig. 5:**
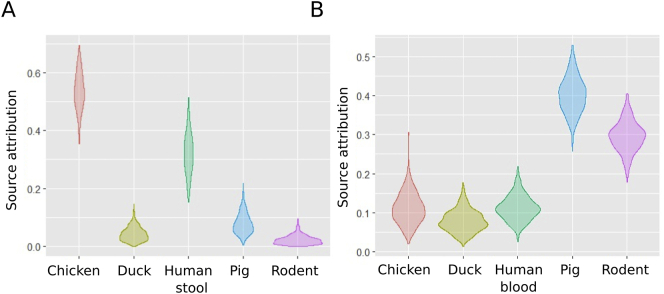
Source attribution of human NTS isolates in Vietnam by ST-AMR profile. Violin plots showing the results of the source attribution model for NTS infections in human blood (A) and human stool (B). Each plot represents the mixture coefficient (α), which is an estimated proportion of NTS cases attributed to each source according to combined ST-MR profile using Bayesian multinomial mixture modelling with sampled sources. The centre of each violin represents the median, the length represents credibility interval and the shape displays frequencies of values. Each of the sampled sources is labelled on the x axis and the proportional contribution (source attribution) is labelled on the y axis.

To investigate the potential role of zoonotic sources in the AMR-NTS phenotypes in humans, we compared the combined ST-AMR profiles of human and animal isolates. We identified 242 unique ST-AMR profiles. When comparing ST-AMR profiles by the source of isolate, 47 profiles were unique to human blood, 114 to animals, and 19 were shared. Among human stool and animal isolates, 67 ST-AMR profiles were found only in human stool only, 90 in animal stool only, and 43 in both (Fig. 4) (Tables S8–10). Modelling the potential source of NTS in human blood by ST-AMR profile found that 54% (95% CrI 39.9–66.1) could be attributed to chickens and 32% (95% CrI 18.6–46.4) to humans with non-invasive disease. For NTS in human stool, the majority could be attributed to pigs (40%; 95%CrI 31.7–49.4) (Fig. 5, Table S11). When allowing for an unsampled source using ST-AMR profile, modelling again identified pigs as the principal contributor for NTS in human blood and human stools (Fig. S6).

## 4 Discussion

Our data show that the structure NTS serovars, STs, and AMR profiles circulating in animals and humans in Vietnam is complex. Notably, AMR in iNTS infections is common, with 81% of blood isolates resistant to at least one antimicrobial agent. This finding is supported by other studies where AMR-NTS infections were more commonly associated with bloodstream infection and had worse clinical outcomes when compared to pan-susceptible organisms (Parisi et al., 2018; Varma et al., 2005). We also demonstrated that NTS infection substantially differs by serovar with serovar Choleraesuis causing bloodstream infection only and serovar Newport primarily causing enterocolitis (Crump et al., 2011; Jones et al., 2008).

There are several potential explanations for such a high prevalence of AMR in human NTS infections in Vietnam. A lack of knowledge about AMU, sales without prescription, inadequate infection control, a lack of adequate microbiology services, and lack of effective drug and therapeutics committees are likely key reasons for the AMU both in the hospitals and community (Global Antibiotic Resistance Partnership Vietnam National Working Group, 2010). A study conducted in 36 hospitals in Vietnam found that 67% of hospitalized patients were treated by antimicrobials; 31% of which were inappropriately indicated (Thu et al., 2012). In the same study, cephalosporins were most commonly prescribed agents and accounted for 70% of all prescriptions.

Antimicrobials are commonly used for growth promotion, prophylaxis, and treatment in farm animals. Antimicrobials account for 70% of all registered drugs used in animal production in Vietnam. A surveillance study conducted in 30 chicken and 30 pig farms found that 60% and 70% of derived products respectively, were contaminated with tetracyclines or tylosins (An, 2009). A further study from Mekong Delta found that 27% of *Salmonella* from retail meat were resistant to at least one antimicrobial agent (Ogasawara et al., 2008). Here, the highest resistance in animal isolates was observed against ampicillin, trimethoprim-sulfamethoxazole, and chloramphenicol with 23% of all isolates resistant to these agents, which is considerably higher than observed previously (Ogasawara et al., 2008).

When comparing ST-AMR profiles, 51% and 60% of human blood and stool isolates, respectively, shared their profile with animal isolates. This finding supports the notion that the AMU in animals may play an important role in the transmission of AMR to humans through food or via direct animal contact (Angulo et al., 2000). However, AMR in animal NTS isolates did not explain the full extent of AMR in invasive human infections and other unsampled sources are important. Consequently, we hypothesize that AMR plasmids that reside in commensal organisms in the human gut are likely a major reservoir for AMR genes that can be transferred into *Salmonella* during infection.

The study has limitations. All animal NTS isolates were collected in Mekong Delta (provinces Tien Giang and Dong Thap) whereas majority of human NTS isolates was collected from the hospitals in HCMC. Although all three provinces are lie in the close proximity, the serovar and ST distribution may vary. Second, differences in NTS isolation methods for animal and human samples may have had an impact on the detection sensitivity and could preclude identification of greater variety of NTS serovars and STs in humans. Lastly, modelling based on ST-AMR profiles might not have such a discriminatory power as other methods including whole genome sequencing which was not performed mainly due to associated costs and required technology.

## 5 Conclusions

AMU in food animals is considered to be a major contributing factor for the emergence of AMR in humans globally. Although we identified animals as a potential reservoir of AMR in human NTS, AMR in animals does not explain the total extent of resistance observed in humans, suggesting alternative sources, such as other organisms in the human gut, play a key role. Given the prevalence of AMR NTS infections in Vietnam, it is critical to conduct more AMR studies from a One Health perspective, aiming to minimize AMU in humans and animal production.

## Ethical approval

Ethical approval for this study was obtained from the Australian National University Human Research Ethics Committee [protocol 2018/229]. The original studies contributing organisms and data for this analysis obtained ethical approval from the Oxford Tropical Research Ethics Committee (OxTREC). Written informed consent to sample animals in in this study was provided by the owner(s) of the animals. Written informed consent was taken from all individuals enrolled in the study, in the case of those aged under 16 years this was provided by a parent or guardian.

## Consent for publication

All authors have seen and approved the final version of this manuscript for publication.

## Availability of data and materials

All data generated or analyzed during this study are included in this published article and its supplementary information files.

## Funding

We would like to acknowledge the financial support provided by the Endeavour Scholarship (AP), Australasian Epidemiological Association (AP), and Wellcome Trust of the United Kingdom (SB) (215515/Z/19/Z). MDK is supported by the 10.13039/501100000925National Health and Medical Research Council career development fellowship (GNT1136112). The funders had no role in the design and conduct of the study; collection, management, analysis, and interpretation of the data; preparation, review, or approval of the manuscript; and decision to submit the manuscript for publication.

## Authors' contributions

Conceptualization: SB Data curation: TLTP, NHTT, JCM, JIC. Formal analysis: AP, TLPP, AM, TJ. Funding acquisition: MDK, AM, SB. Investigation: AP, TLTP, AM, TJ, HHT, NPHL, NHTT, JCM, JIC, NVT, KG, MDK, SB. Methodology: AP, TLTP, AM, TJ. Project administration: SB. Supervision: MDK, SB. Visualization: AP, TLTP, AM, TJ. Writing - original draft: AP, SB. Writing - review & editing: AP, TLTP, AM, TJ, HHT, NPHL, NHTT, JCM, JIC, NVT, KG, MDK, SB.

## Declaration of Competing Interest

The authors declare that they have no known competing financial interests or personal relationships that could have appeared to influence the work reported in this paper.

## Appendix A The following are the supplementary data related to this article

Appendix 1. Bayesian multinomial mixture model description. Table S1. Characteristics of the studies included in our analyses. Table S2. The distribution of NTS serovars and STs in human blood. Table S3. The distribution of NTS serovars and STs in human stool. Table S4. The distribution of NTS serovars and STs in animals. Table S5. The distribution of AMR profiles in human blood NTS isolates. Table S6. The distribution of AMR profiles in human stool NTS isolates. Table S7. The distribution of AMR profiles in animal NTS isolates. Table S8. The distribution of ST-AMR profiles in human blood NTS isolates. Table S9. The distribution of ST-AMR profiles in human stool NTS isolates. Table S10. The distribution of ST-AMR profiles in animal NTS isolates. Table S11. Source attribution models of NTS in human blood and human stool. Fig. S1. Distribution of sequence types by the source of isolate Each column corresponds to different source and the size of the circle corresponds to the number of isolates per sequence type. Fig. S2. Source attribution of human NTS isolates in Vietnam by STs Violin plots showing the results of the source attribution model for NTS infections in human blood (A) and human stool (B). Each plot represents the mixture coefficient (α), which is an estimated proportion of NTS cases attributed to each source according to sequence type using Bayesian multinomial mixture modelling with sampled and unsampled sources. The centre of each violin represents the median, the length represents credibility interval and the shape displays frequencies of values. Each of the sampled sources is labelled on the x axis and the proportional contribution (source attribution) is labelled on the y axis. Fig. S3. Distribution of AMR profiles by the source of isolate Each column corresponds to different source and the size of the circle corresponds to the number of isolates per antimicrobial susceptibility profile (AMR). Fig. S4. Number and proportion of unique and shared AMR profiles Venn diagrams showing the number of unique and shared AMR profiles between organisms isolated from human blood and animals (A) and human stools and animals (C). Pie charts showing the unique and shared AMR profiles between organisms isolated from human blood and animals (B) and human stools and animals (D). Fig. S5. Source attribution of NTS isolates in humans by AMR profile Violin plots showing the results of the source attribution model for NTS infections in human blood (A) and human stool (B). Each plot represents the mixture coefficient (α), which is an estimated proportion of NTS cases attributed to each source according to AMR profile using Bayesian multinomial mixture modelling with sampled and unsampled sources. The centre of each violin represents the median, the length represents credibility interval and the shape displays frequencies of values. Each of the sampled sources is labelled on the x axis and the proportional contribution (source attribution) is labelled on the y axis. Fig. S6. Source attribution of NTS isolates in humans by ST-AMR profile Violin plots showing the results of the source attribution model for NTS infections in human blood (A) and human stool (B). Each plot represents the mixture coefficient (α), which is an estimated proportion of NTS cases attributed to each source according to combined ST-MR profile using Bayesian multinomial mixture modelling with sampled and unsampled sources. The centre of each violin represents the median, the length represents credibility interval and the shape displays frequencies of values. Each of the sampled sources is labelled on the x axis and the proportional contribution (source attribution) is labelled on the y axis. Image 1
