# Supplementary material for: The role of animals as a source of antimicrobial resistant nontyphoidal Salmonella causing invasive and non-invasive human disease in Vietnam
Source: Infect Genet Evol. 2020 Nov;85:104534. doi: 10.1016/j.meegid.2020.104534 (PMC7705210; doi:10.1016/j.meegid.2020.104534)
Supplement: Supplementary file 1 — Appendix 1. Bayesian multinomial mixture model description. Table S1. Characteristics of the studies included in our analyses. Table S2. The distribution of NTS serovars and STs in human blood. Table S3. The distribution of NTS serovars and STs in human stool. Table S4. The distribution of NTS serovars and STs in animals. Table S5. The distribution of AMR profiles in human blood NTS isolates. Table S6. The distribution of AMR profiles in human stool NTS isolates. Table S7. The distribution of AMR profiles in animal NTS isolates. Table S8. The distribution of ST-AMR profiles in human blood NTS isolates. Table S9. The distribution of ST-AMR profiles in human stool NTS isolates. Table S10. The distribution of ST-AMR profiles in animal NTS isolates. Table S11. Source attribution models of NTS in human blood and human stool. Fig. S1. Distribution of sequence types by the source of isolate Each column corresponds to different source and the size of the circle corresponds to the number of isolates per sequence type. Fig. S2. Source attribution of human NTS isolates in Vietnam by STs Violin plots showing the results of the source attribution model for NTS infections in human blood (A) and human stool (B). Each plot represents the mixture coefficient (α), which is an estimated proportion of NTS cases attributed to each source according to sequence type using Bayesian multinomial mixture modelling with sampled and unsampled sources. The centre of each violin represents the median, the length represents credibility interval and the shape displays frequencies of values. Each of the sampled sources is labelled on the x axis and the proportional contribution (source attribution) is labelled on the y axis. Fig. S3. Distribution of AMR profiles by the source of isolate Each column corresponds to different source and the size of the circle corresponds to the number of isolates per antimicrobial susceptibility profile (AMR). Fig. S4. Number and proportion of unique and shared AMR pro [file mmc1.docx]

**Supplementary Information**

**Appendix 1. Bayesian multinomial mixture model description**

Our model describes the empirical distribution of isolates of different groups (STs, AMRs profiles) *y* as a mixture of *K* different sampled origins noted *x*_1_, *x*_2_, ..., *x_K_* with corresponding mixture coefficient  *α*_1_, *α*_2_, ..., *α_K_* (with *α*_1_ + *α*_2_ + ... + *α_K_* = 1). These coefficients are weights describing the contributions of each origin to the observed distribution of interest. We noted *n*_1_, *n*_2_, ..., *n_K_* the sample sizes of the origins, and *n* the sample size of *y*. Assuming that all origins contribute independently to *y*, the distribution of *y* is defined as:

*y* ~ Multinomial(*α*_1_ *φ*_1_, *α_2_* *φ*_2_, ..., *α_K_* *φ_K_*, *n*)

where *φ_i_* is the frequency distribution of the groups in origin *i*. Similiary, the empirical distribution within any origin *i* is defined as:

*x_i_* ~ Multinomial(*φ_i_*, *n_i_*)

Given these distributions, we can write the posterior distribution of our model as:

*p*(y, *x*_1_, *x*_2_, ..., *x_K_* | *α*_1_ *φ*_1_, *α_2_* *φ*_2_, ..., *α_K_* *φ_K_*) =

( *p*(y, *x*_1_, *x*_2_, ..., *x_K_* | *α*_1_ *φ*_1_, *α_2_* *φ*_2_, ..., *α_K_* *φ_K_*) *p*(*α*_1_ *φ*_1_, *α_2_* *φ*_2_, ..., *α_K_* *φ_K_*) ) / *p*(y, *x*_1_, *x*_2_, ..., *x_K_*)

where *p*(y, *x*_1_, *x*_2_, ..., *x_K_*) is a constant representing the marginal probability of the data. This posterior distribution is then proportional to:

*p*(y, *x*_1_, *x*_2_, ..., *x_K_* | *α*_1_ *φ*_1_, *α_2_* *φ*_2_, ..., *α_K_* *φ_K_*) *p*(*α*_1_ *φ*_1_, *α_2_* *φ*_2_, ..., *α_K_* *φ_K_*)

where the first term is the likelihood of our model, and the second term represents the priors. The likelihood is written as a product of independent terms:

*p*(y, *x*_1_, *x*_2_, ..., *x_K_* | *α*_1_ *φ*_1_, *α_2_* *φ*_2_, ..., *α_K_* *φ_K_*) =

*p*(y | *α*_1_ *φ*_1_, *α_2_* *φ*_2_, ..., *α_K_* *φ_K_*) *p*(*x*_1_ | *α*_1_ *φ*_1_) *p*(*x*_2_ | *α_2_* *φ*_2_) ... *p*(*x_K_* | *α_K_* *φ_K_*) =

*F_M_*(y, *α*_1_ *φ*_1_, *α_2_* *φ*_2_, ..., *α_K_* *φ_K,_ n*) *F_M_*(*x*_1_, *φ*_1_, *n*_1_) *F_M_*(*x*_2_, *φ_2_*, *n*_2_) ... *F_M_*(*x_K_*, *φ_K_*, *n_K_*)

where *F_M_* refers to the Multinomial probability mass function.

The joint prior distribution is rewritten as a product of independent components:

*p*(*α*_1_ *φ*_1_, *α_2_* *φ*_2_, ..., *α_K_* *φ_K_*) =

*p*(*α*_1_, *α_2_* , ..., *α_K_*) *p*(*φ*_1_, *φ*_2_, ..., *φ_K_*) =

*p*(*α*_1_, *α_2_* , ..., *α_K_*) *p*(*φ*_1_) *p*(*φ*_2_) ... *p*(*φ_K_*)

We assume no prior information on the mixture coefficients or the group frequencies so that these priors can be modelled by a constant Dirichlet distribution, and are effectively treated as constants in the estimation procedure.

The model above can be easily extended to incorporate an *unsampled* origin, by adding a new origin *x_unsampled_* containing only 0s and with sample size *n_unsampled_* = 0, and frequencies *φ_unsampled_*.

**Estimation**

Markov Chain Monte Carlo can be used to derive samples of the parameters from the posterior distribution. We use a simple Metropolis algorithm for all parameters, using Dirichlet proposals for the mixture coefficients and for the group frequencies. In theory, both the contribution coefficients and the frequencies could be estimated simultaneously. However, for computational efficiency frequencies of groups in the different origins *φ_i_* can also be fixed to their Maximum Likelihood estimates so that *φ_i_* = *x_i_* / *n_i_.* Note that if the model includes an unsampled origin, group frequencies *φ_unsampled_* have to be estimated, whether or not other frequency distributions *φ_i_* are estimated or fixed. Convergence is assessed by ensuring stationarity of the different parameters from visual inspection of their traces.

**Availability**

The Bayesian Mixture model has been implemented in the R package *bmmix* available from CRAN and github:

CRAN: <https://cran.r-project.org/web/packages/bmmix/index.html>

github (development version): <https://github.com/thibautjombart/bmmix>

*bmmix* is distributed under Gnu General Public License (GPL) ≥ 2.

| **Study name** | **Period** | **Location** | **Study type** | **Study population** | **Study aims** |
| --- | --- | --- | --- | --- | --- |
| The Vietnam Initiative on Zoonotic Infections (VIZIONs). | Not specified (5 years of retrospective and 5 years of prospective data from provincial hospitals) and high risk-cohorts. | Provincial hospitals in Dong Thap, Ho ChiMinh City, Dak Lak, Khanh Hoa, Hue, Hanoi, and Ba Vi; and high risk cohorts in Dong Thap, Dak  Lak, and Ba Vi. | Hospital-based surveillance program + longitudinal cohort study | 1) Disease surveillance in provincial hospitals: ongoing enrollment of patients (>12,000 over 5 years) with diarrhea, respiratory tract infections, aundice/hepatitis, and central nervous system infections.  2) High-risk cohort and social studies: Animal baseline sampling in three provinces; enrolment of 852 cohort members (farmers, animal health workers, butchers, market traders); follow-up sampling every 12 months. | 1. To establish international collaborative consortium with an integrated approach to human and animal health research;  2. To estimate the burden of zoonotic diseases disease and investigate the disease epidemiology in hospitalized patients and individuals occupationally exposed to animals;  3. To characterize genetic diversity and describe disease transmission;  4. To identify socio-demographic, environmental, and behavioral drivers for disease emergence. |
| The bacterial etiology and antimicrobial susceptibility profile of bloodstream infections at the Hospital for Tropical Diseases in Ho Chi Minh City. | January 2010-December 2013 | Hospital for Tropical Diseases (HTD), Ho Chi Minh City, Vietnam | Retrospective hospital-based study | Patients at HTD with a positive bacterial culture from a blood sample from the 1st January 2010 to 31st December 2013 | To understand the main causes of bacteremia and their associated antimicrobial susceptibility profiles at HTD. |
| The aetiology diarrheal disease in children in Hoi Chi Minh City | May 2009-April 2010 | Children’s Hospital 1 (CH1), Children’s Hospital 2 (CH2), and the Hospital for Tropical Diseases (HTD), Ho Chi Minh City, Vietnam | Prospective hospital-based study | Patients at CH1, CH2, and HTD with a positive bacterial culture from stool | To understand the aetiology of diarrhea in Southern Vietnam |
| The Vietnam Bacterial Resistance (VIBRE). | March 2012 to April 2013 | Tien Giang | Cross-sectional study | 204 backyard chicken farms, 204 farmers and 306 matched individuals not exposed to chicken farming. | To investigate the risk of NTS colonization in humans as a result of direct animal exposure. |

**Table S1.** Characteristics of the studies included in our analyses

**Table S2.** The distribution of NTS serovars and corresponding sequence types among human blood isolates in Vietnam.

| Serovar/ Sequence type | No. | % |
| --- | --- | --- |
| Enteritidis | 62 | 41.89 |
| ST 11 | 62 | 100 |
| Typhimurium | 44 | 29.73 |
| ST 34 | 28 | 63.64 |
| ST 19 | 14 | 31.82 |
| ST 1544 | 2 | 4.55 |
| Choleraesuis | 15 | 10.14 |
| ST 68 | 14 | 93.33 |
| ST 139 | 1 | 6.67 |
| Paratyphi B var Java | 3 | 2.03 |
| ST 42 | 1 | 33.33 |
| ST 135 | 1 | 33.33 |
| ST 423 | 1 | 33.33 |
| Rissen | 3 | 2.03 |
| ST 469 | 3 | 100 |
| Stanley | 3 | 2.03 |
| ST 29 | 3 | 100 |
| Corvallis | 2 | 1.35 |
| ST 1541 | 2 | 100 |
| Give | 2 | 1.35 |
| ST 516 | 2 | 100 |
| Sub species IV | 2 | 1.35 |
| ST 958 | 2 | 100 |
| Albany | 1 | 0.68 |
| ST 292 | 1 | 100 |
| Brandenburg | 1 | 0.68 |
| ST 20 | 1 | 100 |
| Derby | 1 | 0.68 |
| ST 40 | 1 | 100 |
| Indiana | 1 | 0.68 |
| ST 17 | 1 | 100 |
| Montevideo | 1 | 0.68 |
| ST 1531 | 1 | 100 |
| Newport | 1 | 0.68 |
| ST 31 | 1 | 100 |
| Schwarzengrund | 1 | 0.68 |
| ST 96 | 1 | 100 |
| Weltevreden | 1 | 0.68 |
| ST 365 | 1 | 100 |
| N/A | 4 | 2.70 |
| ST 48 | 2 | 50 |
| ST 1864 | 1 | 25 |
| ST 1873 | 1 | 25 |
| **Total** | **148** | **100** |

**Table S3.** The distribution of NTS serovars and corresponding sequence types among human stool isolates in Vietnam.

| Serovar/Sequence type | No. | % |  |
| --- | --- | --- | --- |
| Typhimurium | 46 | 21.8 |  |
| ST 19 | 21 | 45.65 |  |
| ST 34 | 20 | 43.48 |  |
| ST 36 | 2 | 4.35 |  |
| ST 99 | 1 | 2.17 |  |
| ST 313 | 1 | 2.17 |  |
| ST 1544 | 1 | 2.17 |  |
| Weltevreden | 41 | 19.43 |  |
| ST 365 | 33 | 80.49 |  |
| ST 1500 | 8 | 19.51 |  |
| Newport | 11 | 5.21 |  |
| ST 46 | 9 | 81.82 |  |
| ST 31 | 2 | 18.18 |  |
| Derby | 10 | 4.74 |  |
| ST 40 | 10 | 100 |  |
| Stanley | 9 | 4.27 |  |
| ST 29 | 8 | 88.89 |  |
| ST 1550 | 1 | 11.11 |  |
| Rissen | 8 | 3.79 |  |
| ST 469 | 8 | 100 |  |
| Enteritidis | 6 | 2.84 |  |
| ST 11 | 6 | 100 |  |
| Litchfield | 5 | 2.37 |  |
| ST 1499 | 3 | 60 |  |
| ST 214 | 2 | 40 |  |
| Corvallis | 4 | 1.9 |  |
| ST 1541 | 4 | 100 |  |
| Paratyphi B var Java monophasic | 4 | 1.9 |  |
| ST 423 | 4 | 100 |  |
| Give | 3 | 1.42 |  |
| ST 516 | 3 | 100 |  |
| Virchow | 3 | 1.42 |  |
| ST 359 | 2 | 66.67 |  |
| ST 197 | 1 | 33.33 |  |
| Anatum | 2 | 0.95 |  |
| ST 64 | 2 | 100 |  |
| Braenderup | 2 | 0.95 |  |
| ST 22 | 2 | 100 |  |
| Bredeney | 2 | 0.95 |  |
| ST 1543 | 2 | 100 |  |
| Hadar | 2 | 0.95 |  |
| ST 33 | 2 | 100 |  |
| Kentucky | 2 | 0.95 |  |
| ST 314 | 2 | 100 |  |
| London | 2 | 0.95 |  |
| ST 155 | 2 | 100 |  |
| Mbandaka | 2 | 0.95 |  |
| ST 413 | 2 | 100 |  |
| Ohio | 2 | 0.95 |  |
| ST 329 | 2 | 100 |  |
| Bareilly | 1 | 0.47 |  |
| ST 203 | 1 | 100 |  |
| Cerro var. Siegburg | 1 | 0.47 |  |
| ST 367 | 1 | 100 |  |
| Indiana | 1 | 0.47 |  |
| ST 17 | 1 | 100 |  |
| Meleagridis | 1 | 0.47 |  |
| ST 463 | 1 | 100 |  |
| Muenchen | 1 | 0.47 |  |
| ST 82 | 1 | 100 |  |
| Orientalis | 1 | 0.47 |  |
| ST 558 | 1 | 100 |  |
| Pomona | 1 | 0.47 |  |
| ST 451 | 1 | 100 |  |
| Rubislaw | 1 | 0.47 |  |
| ST 820 | 1 | 100 |  |
| Saintpaul | 1 | 0.47 |  |
| ST 50 | 1 | 100 |  |
| Subspecies IIIb | 1 | 0.47 |  |
| ST 430 | 1 | 100 |  |
| Worthington | 1 | 0.47 |  |
| ST 592 | 1 | 100 |  |
| N/A | 34 | 16.11 |  |
| ST 1542 | 10 | 29.41 |  |
| ST 292 | 7 | 20.59 |  |
| ST 48 | 3 | 8.82 |  |
| ST 74 | 2 | 5.88 |  |
| ST 1546 | 2 | 5.88 |  |
| ST 909 | 1 | 2.94 |  |
| ST 1545 | 1 | 2.94 |  |
| ST 1547 | 1 | 2.94 |  |
| ST 1548 | 1 | 2.94 |  |
| ST 1549 | 1 | 2.94 |  |
| ST 1561 | 1 | 2.94 |  |
| ST 1562 | 1 | 2.94 |  |
| ST 1867 | 1 | 2.94 |  |
| ST 1871 | 1 | 2.94 |  |
| ST 1874 | 1 | 2.94 |  |
| **Total** | **211** | **100** |  |

**Table S4.** The distribution of NTS serovars and corresponding sequence types among NTS animal isolates in Vietnam.

| **Serovar/ Sequence type** | **No.** | **%** |
| --- | --- | --- |
| Weltevreden | 43 | 13.74 |
| ST 1500 | 41 | 95.35 |
| ST 365 | 2 | 4.65 |
| Typhimurium | 32 | 10.22 |
| ST 1544 | 14 | 43.75 |
| ST 19 | 9 | 28.13 |
| ST 34 | 7 | 21.88 |
| ST 36 | 2 | 6.25 |
| Paratyphi B var Java monophasic | 26 | 8.31 |
| ST 423 | 13 | 50 |
| ST 42 | 8 | 30.77 |
| ST 135 | 4 | 15.38 |
| ST 43 | 1 | 3.85 |
| Indiana | 18 | 5.75 |
| ST 17 | 18 | 100 |
| Newport | 17 | 5.43 |
| ST 31 | 11 | 64.71 |
| ST 46 | 6 | 35.29 |
| Enteritidis | 16 | 5.11 |
| ST 11 | 14 | 87.5 |
| ST 180 | 1 | 6.25 |
| ST 1863 | 1 | 6.25 |
| Derby | 14 | 4.47 |
| ST 40 | 14 | 100 |
| Rissen | 10 | 3.19 |
| ST 469 | 10 | 100 |
| Anatum | 8 | 2.56 |
| ST 64 | 8 | 100 |
| Hadar | 8 | 2.56 |
| ST 33 | 8 | 100 |
| Oslo | 8 | 2.56 |
| ST 1370 | 8 | 100 |
| Give | 7 | 2.24 |
| ST 516 | 7 | 100 |
| Kentucky | 7 | 2.24 |
| ST 314 | 6 | 85.71 |
| ST 1865 | 1 | 14.29 |
| Meleagridis | 6 | 1.92 |
| ST 463 | 6 | 100 |
| London | 5 | 1.6 |
| ST 155 | 5 | 100 |
| Albany | 4 | 1.28 |
| ST 292 | 4 | 100 |
| Senftenberg | 4 | 1.28 |
| ST 14 | 4 | 100 |
| Stanley | 4 | 1.28 |
| ST 29 | 2 | 50 |
| ST 182 | 2 | 50 |
| Tennessee | 4 | 1.28 |
| ST 319 | 4 | 100 |
| Braenderup | 3 | 0.96 |
| ST 22 | 3 | 100 |
| Litchfield | 3 | 0.96 |
| ST 1499 | 3 | 100 |
| Sandiego | 3 | 0.96 |
| ST 1568 | 3 | 100 |
| Virchow | 3 | 0.96 |
| ST 359 | 3 | 100 |
| Bareilly | 2 | 0.64 |
| ST 203 | 2 | 100 |
| Corvallis | 2 | 0.64 |
| ST 1541 | 2 | 100 |
| Bovismorbificans | 1 | 0.32 |
| ST 1058 | 1 | 100 |
| Heidelberg | 1 | 0.32 |
| ST 15 | 1 | 100 |
| Mbandaka | 1 | 0.32 |
| ST 413 | 1 | 100 |
| Saintpaul | 1 | 0.32 |
| ST 50 | 1 | 100 |
| Tananarive | 1 | 0.32 |
| ST 1794 | 1 | 100 |
| Urbana | 1 | 0.32 |
| ST 512 | 1 | 100 |
| N/A | 50 | 15.97 |
| ST 1547 | 13 | 26 |
| ST 74 | 9 | 18 |
| ST 1546 | 9 | 18 |
| ST 1562 | 7 | 14 |
| ST 292 | 4 | 8 |
| ST 1861 | 3 | 6 |
| ST 1867 | 3 | 6 |
| ST 1866 | 1 | 2 |
| ST 1868 | 1 | 2 |
| **Total** | **313** | **100** |

**Table S5.** Distribution of 40 antimicrobial susceptibility profiles among human blood NTS isolates in Vietnam.

| Antimicrobial susceptibility profile^a^ | No. of antimicrobial agents | No. of isolates | % |
| --- | --- | --- | --- |
| Fully susceptible | 0 | 28 | 18.92 |
| Gen | 1 | 1 | 0.68 |
| Cip | 1 | 18 | 12.16 |
| Amp | 1 | 19 | 12.84 |
| ChlCip | 2 | 2 | 1.35 |
| CtxCip | 2 | 1 | 0.68 |
| AmpSxt | 2 | 7 | 4.73 |
| AmpGen | 2 | 1 | 0.68 |
| AmpCip | 2 | 7 | 4.73 |
| AmpChl | 2 | 3 | 2.03 |
| AmpGenSxt | 3 | 1 | 0.68 |
| AmpCpSxt | 3 | 1 | 0.68 |
| AmpChlSxt | 3 | 4 | 2.7 |
| AmpChlGen | 3 | 1 | 0.68 |
| AmpChlCip | 3 | 3 | 2.03 |
| AmpCipGenSxt | 4 | 4 | 2.7 |
| AmpChlGenSxt | 4 | 3 | 2.03 |
| AmpChlCipSxt | 4 | 4 | 2.7 |
| AmpChlCipGen | 4 | 2 | 1.35 |
| AmpAkChlSxt | 4 | 1 | 0.68 |
| AmpAkChlCip | 4 | 2 | 1.35 |
| AmpChlCipGenSxt | 5 | 16 | 10.81 |
| AmpAkCipGenSxt | 5 | 1 | 0.68 |
| AmpAkChlGenSxt | 5 | 2 | 1.35 |
| AmpAkChlCipSxt | 5 | 2 | 1.35 |
| AmpAkChlCipGen | 5 | 2 | 1.35 |
| AmpCfzCtxChlGenSxt | 6 | 1 | 0.68 |
| AmpAkChlCipGenSxt | 6 | 9 | 6.08 |
| AmpAkCtxChlCipGenSxt | 7 | 1 | 0.68 |
| AmpAkCfzCtxChlCipGenSxt | 8 | 1 | 0.68 |
| **Total** |  | **148** | **100** |

^a^Abbreviated antimicrobial agents: Amp, ampicillin; Ak, amikacin; Caz, ceftazidime, Ctx, ceftriaxone; Chl, chloramphenicol, Cip, ciprofloxacin; Gen, gentamicin; Stx, trimethoprim-sulfamethoxazole

**Table S6.** The distribution of 30 antimicrobial susceptibility profiles among human stool NTS isolates in Vietnam.

| **Antimicrobial susceptibility profile^a^** | **No. of antimicrobial agents** | **No. of isolates** | **%** |
| --- | --- | --- | --- |
| Fully susceptible | 0 | 111 | 52.61 |
| Sxt | 1 | 8 | 3.79 |
| Gen | 1 | 5 | 2.37 |
| Cip | 1 | 6 | 2.84 |
| Chl | 1 | 5 | 2.37 |
| Amp | 1 | 16 | 7.58 |
| CipSxt | 2 | 1 | 0.47 |
| ChlSxt | 2 | 1 | 0.47 |
| AkChl | 2 | 1 | 0.47 |
| AmpSxt | 2 | 4 | 1.9 |
| AmpAk | 2 | 3 | 1.42 |
| ChlCipSxt | 3 | 1 | 0.47 |
| CfzChlCip | 3 | 1 | 0.47 |
| AmpGenSxt | 3 | 2 | 0.95 |
| AmpChlSxt | 3 | 16 | 7.58 |
| AmpChlGen | 3 | 3 | 1.42 |
| AmpChlGenSxt | 4 | 7 | 3.32 |
| AmpChlCipSxt | 4 | 1 | 0.47 |
| AmpAkGenSxt | 4 | 1 | 0.47 |
| AmkAkChlSxt | 4 | 3 | 1.42 |
| AmpAkChlGen | 4 | 2 | 0.95 |
| AmkAkChlCip | 4 | 1 | 0.47 |
| AmpAkCfzCtx | 4 | 2 | 0.95 |
| AmpChlCipGenSxt | 5 | 2 | 0.95 |
| AmpAkChlGenSxt | 5 | 1 | 0.47 |
| AmkAkChlCipSxt | 5 | 1 | 0.47 |
| AmkAkChlCipGen | 5 | 2 | 0.95 |
| AmkAkCfzCtxGenSxt | 6 | 1 | 0.47 |
| AmkAkCfzCtxChlSxt | 6 | 2 | 0.95 |
| AmpAkCfzCtxChlCipGenSxt | 8 | 1 | 0.47 |
| **Total** |  | **211** | **100** |

^a^Abbreviated antimicrobial agents: Amp, ampicillin; Ak, amikacin; Caz, ceftazidime, Ctx, ceftriaxone; Chl, chloramphenicol, Cip, ciprofloxacin; Gen, gentamicin; Stx, trimethoprim-sulfamethoxazole

**Table S7.** The distribution of 34 antimicrobial susceptibility profiles among animal NTS isolates in Vietnam.

| Antimicrobial susceptibility profile^a^ | No. of antimicrobial agents | No. of isolates | % |
| --- | --- | --- | --- |
| Fully susceptible | 0 | 167 | 53.35 |
| Sxt | 1 | 10 | 3.19 |
| Gen | 1 | 3 | 0.96 |
| Cip | 1 | 29 | 9.27 |
| Chl | 1 | 8 | 2.56 |
| Cfz | 1 | 1 | 0.32 |
| Ak | 1 | 1 | 0.32 |
| Amp | 1 | 15 | 4.79 |
| CipSxt | 2 | 1 | 0.32 |
| CipGen | 2 | 1 | 0.32 |
| ChlSxt | 2 | 14 | 4.47 |
| ChlCip | 2 | 1 | 0.32 |
| CtxChl | 2 | 1 | 0.32 |
| AkCip | 2 | 1 | 0.32 |
| AmpSxt | 2 | 4 | 1.28 |
| AmpCip | 2 | 2 | 0.64 |
| AmpChl | 2 | 3 | 0.96 |
| AmpAk | 2 | 4 | 1.28 |
| ChlCipSxt | 3 | 1 | 0.32 |
| AkChlSxt | 3 | 1 | 0.32 |
| AkChlCip | 3 | 1 | 0.32 |
| AmpGenSxt | 3 | 1 | 0.32 |
| AmpCipSxt | 3 | 1 | 0.32 |
| AmpChlSxt | 3 | 12 | 3.83 |
| AmpChlGenSxt | 4 | 3 | 0.96 |
| AmpChlCipSxt | 4 | 9 | 2.88 |
| AmpAkCipSxt | 4 | 1 | 0.32 |
| AmpAkChlSxt | 4 | 2 | 0.64 |
| AmpChlCipGenSxt | 5 | 5 | 1.6 |
| AmpAkChlCipSxt | 5 | 4 | 1.28 |
| AmpAkChlCipGen | 5 | 1 | 0.32 |
| AmpAkCfzCtxChl | 5 | 1 | 0.32 |
| AmpAkChlCipGenSxt | 6 | 3 | 0.96 |
| AmpAkCfzCtxChlGen | 6 | 1 | 0.32 |
| **Total** |  | **313** | **100** |

^a^Abbreviated antimicrobial agents: Amp, ampicillin; Ak, amikacin; Caz, ceftazidime, Ctx, ceftriaxone; Chl, chloramphenicol, Cip, ciprofloxacin; Gen, gentamicin; Stx, trimethoprim-sulfamethoxazole

**Table S8.** The distribution of 66 ST-AMR profiles among human blood NTS isolates in Vietnam.

| ST_Antimicrobial susceptibility profile^a^ | No. of antimicrobial agents | No. of isolates | % |
| --- | --- | --- | --- |
| 11_Fully susceptible | 0 | 19 | 12.84 |
| 11_Cip | 1 | 9 | 6.08 |
| 11_Amp | 1 | 18 | 12.16 |
| 11_AmpSxt | 2 | 5 | 3.38 |
| 11_AmpGen | 2 | 1 | 0.68 |
| 11_AmpCip | 2 | 5 | 3.38 |
| 11_AmpChl | 2 | 1 | 0.68 |
| 11_AmpCipSxt | 3 | 1 | 0.68 |
| 11_AmpChlSxt | 3 | 1 | 0.68 |
| 11_AmpChlCip | 3 | 2 | 1.35 |
| 17_AmpAkChlCipGenSxt | 6 | 1 | 0.68 |
| 19_Fully susceptible | 0 | 2 | 1.35 |
| 19_Gen | 1 | 1 | 0.68 |
| 19_AmpCipGenSxt | 4 | 2 | 1.35 |
| 19_AmpChlCipSxt | 4 | 3 | 2.03 |
| 19_AmpAkChlCip | 4 | 2 | 1.35 |
| 19_AmpAkChlSxt | 4 | 1 | 0.68 |
| 19_AmpChlCipGen | 4 | 1 | 0.68 |
| 19_AmpChlCipGenSxt | 5 | 1 | 0.68 |
| 19_AmpAkCipGenSxt | 5 | 1 | 0.68 |
| 20_Fully susceptible | 0 | 1 | 0.68 |
| 29_Fully susceptible | 0 | 1 | 0.68 |
| 29_Cip | 1 | 1 | 0.68 |
| 29_Amp | 1 | 1 | 0.68 |
| 31_CtxCip | 2 | 1 | 0.68 |
| 34_Cip | 1 | 1 | 0.68 |
| 34_ChlCip | 2 | 1 | 0.68 |
| 34_AmpCip | 2 | 2 | 1.35 |
| 34_AmpChl | 2 | 1 | 0.68 |
| 34_AmpChlSxt | 3 | 1 | 0.68 |
| 34_AmpChlCip | 3 | 1 | 0.68 |
| 34_AmpChlGen | 3 | 1 | 0.68 |
| 34_AmpChlGenSxt | 4 | 1 | 0.68 |
| 34_AmpChlCipGen | 4 | 1 | 0.68 |
| 34_AmpChlCipGenSxt | 5 | 5 | 3.38 |
| 34_AmpAkChlGenSxt | 5 | 2 | 1.35 |
| 34_AmpAkChlCipSxt | 5 | 1 | 0.68 |
| 34_AmpAkChlCipGen | 5 | 2 | 1.35 |
| 34_AmpAkChlCipGenSxt | 6 | 7 | 4.73 |
| 34_AmpAkCtxChlCipGenSxt | 7 | 1 | 0.68 |
| 40_AmpChl | 2 | 1 | 0.68 |
| 42_Cip | 1 | 1 | 0.68 |
| 48_AmpChlGenSxt | 4 | 1 | 0.68 |
| 48_AmpChlCipGenSxt | 5 | 1 | 0.68 |
| 68_AmpChlSxt | 3 | 1 | 0.68 |
| 68_AmpGenSxt | 3 | 1 | 0.68 |
| 68_AmpCipGenSxt | 4 | 2 | 1.35 |
| 68_AmpChlGenSxt | 4 | 1 | 0.68 |
| 68_AmpChlCipGenSxt | 5 | 8 | 5.41 |
| 68_AmpAkCfzCtxChlCipGenSxt | 8 | 1 | 0.68 |
| 96_AmpChlCipGenSxt | 5 | 1 | 0.68 |
| 135_Gen | 1 | 1 | 0.68 |
| 139_AmpCfzCtxChlGenSxt | 6 | 1 | 0.68 |
| 292_AmpAkChlCipSxt | 5 | 1 | 0.68 |
| 365_Cip | 1 | 1 | 0.68 |
| 423_Cip | 1 | 1 | 0.68 |
| 469_AmpSxt | 2 | 2 | 1.35 |
| 469_AmpChlSxt | 3 | 1 | 0.68 |
| 516_ChlCip | 2 | 1 | 0.68 |
| 516_AmpChlCipSxt | 4 | 1 | 0.68 |
| 958_Fully susceptible | 0 | 2 | 1.35 |
| 1531_Cip | 1 | 1 | 0.68 |
| 1541_Cip | 1 | 2 | 1.35 |
| 1544_Fully susceptible | 0 | 2 | 1.35 |
| 1864_Fully susceptible | 0 | 1 | 0.68 |
| 1873_AmpAkChlCipGenSxt | 6 | 1 | 0.68 |
| **Total** |  | **148** | **100** |

^a^Abbreviated antimicrobial agents: Amp, ampicillin; Ak, amikacin; Caz, ceftazidime, Ctx, ceftriaxone; Chl, chloramphenicol, Cip, ciprofloxacin; Gen, gentamicin; Stx, trimethoprim-sulfamethoxazole

**Table S9.** The distribution of 110 ST-AMR profiles among human stool NTS isolates in Vietnam.

| ST_Antimicrobial susceptibility profile^a^ | No. of antimicrobial agents | No. of isolates | % |
| --- | --- | --- | --- |
| 11_Fully susceptible | 0 | 1 | 0.47 |
| 11_Amp | 1 | 5 | 2.37 |
| 17_AmpAkChlCipGen | 5 | 1 | 0.47 |
| 19_Fully susceptible | 0 | 3 | 1.42 |
| 19_Sxt | 1 | 2 | 0.95 |
| 19_Chl | 1 | 1 | 0.47 |
| 19_Amp | 1 | 1 | 0.47 |
| 19_AmpSxt | 2 | 2 | 0.95 |
| 19_AmpGenSxt | 3 | 1 | 0.47 |
| 19_AmpChlSxt | 3 | 7 | 3.32 |
| 19_AmpChlGenSxt | 4 | 1 | 0.47 |
| 19_AmpAkChlGen | 4 | 1 | 0.47 |
| 19_AmpAkChlCipSxt | 5 | 1 | 0.47 |
| 19_AmpAkCfzCtxGenSxt | 6 | 1 | 0.47 |
| 22_CipSxt | 2 | 1 | 0.47 |
| 22_AmpAk | 2 | 1 | 0.47 |
| 29_Fully susceptible | 0 | 5 | 2.37 |
| 29_Gen | 1 | 1 | 0.47 |
| 29_AmpChlSxt | 3 | 2 | 0.95 |
| 31_Fully susceptible | 0 | 1 | 0.47 |
| 31_ChlCipSxtCtx | 4 | 1 | 0.47 |
| 33_AmpAk | 2 | 2 | 0.95 |
| 34_Fully susceptible | 0 | 1 | 0.47 |
| 34_Amp | 1 | 8 | 3.79 |
| 34_AmpChlSxt | 3 | 1 | 0.47 |
| 34_AmpChlGen | 3 | 1 | 0.47 |
| 34_AmpAkChlGen | 4 | 1 | 0.47 |
| 34_AmpAkCfzCtx | 4 | 1 | 0.47 |
| 34_AmpChlGenSxt | 4 | 2 | 0.95 |
| 34_AmpChlCipGenSxt | 5 | 1 | 0.47 |
| 34_AmpAkChlGenSxt | 5 | 1 | 0.47 |
| 34_AmpAkChlCipGen | 5 | 1 | 0.47 |
| 34_AmpAkCfzCtxChlSxt | 6 | 1 | 0.47 |
| 34_AmpAkCfzCtxChlCipGenSxt | 8 | 1 | 0.47 |
| 36_Fully susceptible | 0 | 2 | 0.95 |
| 40_Fully susceptible | 0 | 3 | 1.42 |
| 40_Chl | 1 | 3 | 1.42 |
| 40_AkChl | 2 | 1 | 0.47 |
| 40_AmpGenSxt | 3 | 1 | 0.47 |
| 40_AmpChlGenSxt | 4 | 1 | 0.47 |
| 40_AmpChlCipGenSxt | 5 | 1 | 0.47 |
| 46_Fully susceptible | 0 | 7 | 3.32 |
| 46_Cip | 1 | 1 | 0.47 |
| 46_AmpAkCfzCtx | 4 | 1 | 0.47 |
| 48_AmpChlGen | 3 | 1 | 0.47 |
| 48_AmpChlGenSxt | 4 | 2 | 0.95 |
| 50_Fully susceptible | 0 | 1 | 0.47 |
| 64_AmpSxt | 2 | 1 | 0.47 |
| 64_AmpAkGenSxt | 4 | 1 | 0.47 |
| 74_Fully susceptible | 0 | 2 | 0.95 |
| 82_Fully susceptible | 0 | 1 | 0.47 |
| 99_Fully susceptible | 0 | 1 | 0.47 |
| 155_Fully susceptible | 0 | 2 | 0.95 |
| 197_Fully susceptible | 0 | 1 | 0.47 |
| 203_Fully susceptible | 0 | 1 | 0.47 |
| 214_Fully susceptible | 0 | 1 | 0.47 |
| 214_AmpChlSxt | 3 | 1 | 0.47 |
| 292_Sxt | 1 | 2 | 0.95 |
| 292_AmpChlSxt | 3 | 1 | 0.47 |
| 292_AmpAkChlSxt | 4 | 3 | 1.42 |
| 292_AmpAkCfzCtxChlSxt | 6 | 1 | 0.47 |
| 313_Fully susceptible | 0 | 1 | 0.47 |
| 314_Fully susceptible | 0 | 2 | 0.95 |
| 329_Cip | 1 | 1 | 0.47 |
| 329_CfzChlCip | 3 | 1 | 0.47 |
| 359_Fully susceptible | 0 | 2 | 0.95 |
| 365_Fully susceptible | 0 | 27 | 12.8 |
| 365_Sxt | 1 | 2 | 0.95 |
| 365_Gen | 1 | 1 | 0.47 |
| 365_Cip | 1 | 1 | 0.47 |
| 365_Amp | 1 | 1 | 0.47 |
| 365_AmpChlSxt | 3 | 1 | 0.47 |
| 367_Gen | 1 | 1 | 0.47 |
| 413_Fully susceptible | 0 | 2 | 0.95 |
| 423_Fully susceptible | 0 | 4 | 1.9 |
| 430_Fully susceptible | 0 | 1 | 0.47 |
| 451_Fully susceptible | 0 | 1 | 0.47 |
| 463_Fully susceptible | 0 | 1 | 0.47 |
| 469_Fully susceptible | 0 | 7 | 3.32 |
| 469_AmpSxt | 2 | 1 | 0.47 |
| 516_Cip | 1 | 1 | 0.47 |
| 516_AmpChlSxt | 3 | 1 | 0.47 |
| 516_AmpChlCipSxt | 4 | 1 | 0.47 |
| 558_Cip | 1 | 1 | 0.47 |
| 592_Sxt | 1 | 1 | 0.47 |
| 820_AmpAkChlCip | 4 | 1 | 0.47 |
| 909_Fully susceptible | 0 | 1 | 0.47 |
| 1499_Fully susceptible | 0 | 1 | 0.47 |
| 1499_Chl | 1 | 1 | 0.47 |
| 1499_AmpChlSxt | 3 | 1 | 0.47 |
| 1500_Fully susceptible | 0 | 6 | 2.84 |
| 1500_Sxt | 1 | 1 | 0.47 |
| 1500_Cip | 1 | 1 | 0.47 |
| 1541_Fully susceptible | 0 | 4 | 1.9 |
| 1542_Fully susceptible | 0 | 9 | 4.27 |
| 1542_Gen | 1 | 1 | 0.47 |
| 1543_AmpChlGen | 3 | 1 | 0.47 |
| 1543_AmpChlGenSxt | 4 | 1 | 0.47 |
| 1544_Fully susceptible | 0 | 1 | 0.47 |
| 1545_Amp | 1 | 1 | 0.47 |
| 1546_Fully susceptible | 0 | 2 | 0.95 |
| 1547_Fully susceptible | 0 | 1 | 0.47 |
| 1548_ChlSxt | 2 | 1 | 0.47 |
| 1549_Fully susceptible | 0 | 1 | 0.47 |
| 1550_AmpChlSxt | 3 | 1 | 0.47 |
| 1561_Fully susceptible | 0 | 1 | 0.47 |
| 1562_Gen | 1 | 1 | 0.47 |
| 1867_Fully susceptible | 0 | 1 | 0.47 |
| 1871_Fully susceptible | 0 | 1 | 0.47 |
| 1874_Fully susceptible | 0 | 1 | 0.47 |
| **Total** |  | **211** | **100** |

^a^Abbreviated antimicrobial agents: Amp, ampicillin; Ak, amikacin; Caz, ceftazidime, Ctx, ceftriaxone; Chl, chloramphenicol, Cip, ciprofloxacin; Gen, gentamicin; Stx, trimethoprim-sulfamethoxazole

**Table S10.** The distribution of 133 ST-AMR profiles among animal NTS isolates in Vietnam.

| ST_Antimicrobial susceptibility profile^a^ | No. of antimicrobial agents | No. of isolates | % |
| --- | --- | --- | --- |
| 11_Fully susceptible | 0 | 7 | 2.24 |
| 11_Cip | 1 | 2 | 0.64 |
| 11_Amp | 1 | 4 | 1.28 |
| 11_AmpCipSxt | 3 | 1 | 0.32 |
| 14_Fully susceptible | 0 | 3 | 0.96 |
| 14_AmpChlGenSxt | 4 | 1 | 0.32 |
| 15_Fully susceptible | 0 | 1 | 0.32 |
| 17_Fully susceptible | 0 | 2 | 0.64 |
| 17_Sxt | 1 | 4 | 1.28 |
| 17_ChlSxt | 2 | 6 | 1.92 |
| 17_AkChlSxt | 3 | 1 | 0.32 |
| 17_AmpAkChlSxt | 4 | 1 | 0.32 |
| 17_AmpChlCipGenSxt | 5 | 3 | 0.96 |
| 17_AmpAkChlCipGenSxt | 6 | 1 | 0.32 |
| 19_Fully susceptible | 0 | 3 | 0.96 |
| 19_AmpSxt | 2 | 1 | 0.32 |
| 19_AmpChl | 2 | 1 | 0.32 |
| 19_AmpChlSxt | 3 | 3 | 0.96 |
| 19_AmpChlGenSxt | 4 | 1 | 0.32 |
| 22_Cip | 1 | 3 | 0.96 |
| 29_Fully susceptible | 0 | 2 | 0.64 |
| 31_Fully susceptible | 0 | 5 | 1.6 |
| 31_Sxt | 1 | 1 | 0.32 |
| 31_Cip | 1 | 2 | 0.64 |
| 31_CipSxt | 2 | 1 | 0.32 |
| 31_ChlSxt | 2 | 1 | 0.32 |
| 31_ChlCipSxtCtx | 4 | 1 | 0.32 |
| 33_Fully susceptible | 0 | 5 | 1.6 |
| 33_Amp | 1 | 1 | 0.32 |
| 33_AmpAk | 2 | 2 | 0.64 |
| 34_Amp | 1 | 2 | 0.64 |
| 34_AmpGenSxt | 3 | 1 | 0.32 |
| 34_AmpChlCipGenSxt | 5 | 1 | 0.32 |
| 34_AmpAkChlCipGen | 5 | 1 | 0.32 |
| 34_AmpAkChlCipGenSxt | 6 | 1 | 0.32 |
| 34_AmpAkCfzCtxChlGen | 6 | 1 | 0.32 |
| 36_Fully susceptible | 0 | 1 | 0.32 |
| 36_AmpChlSxt | 3 | 1 | 0.32 |
| 40_Fully susceptible | 0 | 5 | 1.6 |
| 40_Chl | 1 | 3 | 0.96 |
| 40_ChlSxt | 2 | 1 | 0.32 |
| 40_AmpChl | 2 | 1 | 0.32 |
| 40_AmpChlSxt | 3 | 1 | 0.32 |
| 40_AmpChlGenSxt | 4 | 1 | 0.32 |
| 40_AmpChlCipSxt | 4 | 1 | 0.32 |
| 40_AmpChlCipGenSxt | 5 | 1 | 0.32 |
| 42_Fully susceptible | 0 | 6 | 1.92 |
| 42_Cip | 1 | 1 | 0.32 |
| 42_CtxChl | 2 | 1 | 0.32 |
| 43_Fully susceptible | 0 | 1 | 0.32 |
| 46_Fully susceptible | 0 | 5 | 1.6 |
| 46_Cip | 1 | 1 | 0.32 |
| 50_Cip | 1 | 1 | 0.32 |
| 64_Fully susceptible | 0 | 2 | 0.64 |
| 64_Chl | 1 | 1 | 0.32 |
| 64_ChlSxt | 2 | 3 | 0.96 |
| 64_AmpSxt | 2 | 1 | 0.32 |
| 64_AmpAkCipSxt | 4 | 1 | 0.32 |
| 74_Fully susceptible | 0 | 8 | 2.56 |
| 74_Cip | 1 | 1 | 0.32 |
| 135_Fully susceptible | 0 | 4 | 1.28 |
| 155_Fully susceptible | 0 | 2 | 0.64 |
| 155_AmpChlSxt | 3 | 3 | 0.96 |
| 180_Cip | 1 | 1 | 0.32 |
| 182_Fully susceptible | 0 | 2 | 0.64 |
| 203_Sxt | 1 | 1 | 0.32 |
| 203_AmpAk | 2 | 1 | 0.32 |
| 292_Sxt | 1 | 1 | 0.32 |
| 292_AmpAk | 2 | 1 | 0.32 |
| 292_AmpChlSxt | 3 | 1 | 0.32 |
| 292_AmpAkChlSxt | 4 | 1 | 0.32 |
| 292_AmpAkChlCipSxt | 5 | 4 | 1.28 |
| 314_Fully susceptible | 0 | 5 | 1.6 |
| 314_AmpChl | 2 | 1 | 0.32 |
| 319_Fully susceptible | 0 | 3 | 0.96 |
| 319_ChlCip | 2 | 1 | 0.32 |
| 359_Fully susceptible | 0 | 3 | 0.96 |
| 365_Fully susceptible | 0 | 1 | 0.32 |
| 365_Cip | 1 | 1 | 0.32 |
| 413_CipGen | 2 | 1 | 0.32 |
| 423_Fully susceptible | 0 | 10 | 3.19 |
| 423_Cip | 1 | 2 | 0.64 |
| 423_Ak | 1 | 1 | 0.32 |
| 463_Fully susceptible | 0 | 6 | 1.92 |
| 469_Fully susceptible | 0 | 5 | 1.6 |
| 469_Chl | 1 | 1 | 0.32 |
| 469_Amp | 1 | 1 | 0.32 |
| 469_AmpSxt | 2 | 2 | 0.64 |
| 469_AmpChlSxt | 3 | 1 | 0.32 |
| 512_Fully susceptible | 0 | 1 | 0.32 |
| 516_Fully susceptible | 0 | 2 | 0.64 |
| 516_Chl | 1 | 1 | 0.32 |
| 516_AkChlCip | 3 | 1 | 0.32 |
| 516_AmpChlCipSxt | 4 | 3 | 0.96 |
| 1058_Fully susceptible | 0 | 1 | 0.32 |
| 1370_Fully susceptible | 0 | 6 | 1.92 |
| 1370_Sxt | 1 | 1 | 0.32 |
| 1370_Cip | 1 | 1 | 0.32 |
| 1499_Fully susceptible | 0 | 2 | 0.64 |
| 1499_Amp | 1 | 1 | 0.32 |
| 1500_Fully susceptible | 0 | 25 | 7.99 |
| 1500_Sxt | 1 | 2 | 0.64 |
| 1500_Cip | 1 | 8 | 2.56 |
| 1500_Cfz | 1 | 1 | 0.32 |
| 1500_Amp | 1 | 1 | 0.32 |
| 1500_ChlSxt | 2 | 1 | 0.32 |
| 1500_AmpCip | 2 | 2 | 0.64 |
| 1500_AmpAkChlCipGenSxt | 6 | 1 | 0.32 |
| 1541_Chl | 1 | 1 | 0.32 |
| 1541_ChlSxt | 2 | 1 | 0.32 |
| 1544_Fully susceptible | 0 | 8 | 2.56 |
| 1544_Gen | 1 | 3 | 0.96 |
| 1544_Amp | 1 | 3 | 0.96 |
| 1546_Fully susceptible | 0 | 1 | 0.32 |
| 1546_Cip | 1 | 3 | 0.96 |
| 1546_AkCip | 2 | 1 | 0.32 |
| 1546_AmpChlCipSxt | 4 | 4 | 1.28 |
| 1547_Fully susceptible | 0 | 11 | 3.51 |
| 1547_Cip | 1 | 1 | 0.32 |
| 1547_AmpChlCipSxt | 4 | 1 | 0.32 |
| 1562_Fully susceptible | 0 | 7 | 2.24 |
| 1568_Amp | 1 | 1 | 0.32 |
| 1568_AmpChlSxt | 3 | 1 | 0.32 |
| 1568_AmpAkCfzCtxChl | 5 | 1 | 0.32 |
| 1794_Amp | 1 | 1 | 0.32 |
| 1861_Fully susceptible | 0 | 2 | 0.64 |
| 1861_ChlSxt | 2 | 1 | 0.32 |
| 1863_Fully susceptible | 0 | 1 | 0.32 |
| 1865_Fully susceptible | 0 | 1 | 0.32 |
| 1866_AmpChlSxt | 3 | 1 | 0.32 |
| 1867_Fully susceptible | 0 | 1 | 0.32 |
| 1867_Cip | 1 | 2 | 0.64 |
| 1868_Fully susceptible | 0 | 1 | 0.32 |
| **Total** |  | **313** | **100** |

^a^Abbreviated antimicrobial agents: Amp, ampicillin; Ak, amikacin; Caz, ceftazidime, Ctx, ceftriaxone; Chl, chloramphenicol, Cip, ciprofloxacin; Gen, gentamicin; Stx, trimethoprim-sulfamethoxazole

**Table S11.** Source attribution models of NTS in human blood and human stool by ST, AMR, and ST-AMR profile

| **1. Model with observed sources** | | | | | | | | | | | | | |
| --- | --- | --- | --- | --- | --- | --- | --- | --- | --- | --- | --- | --- | --- |
|  | **Human blood (n=148)** | | | | |  |  | **Human stool (n=211)** | | | | |  |
|  | Mixture coefficient α (x 100)^a^  (95% CrI)^b^ | | | | |  |  | Mixture coefficient α (x 100)^a^  (95% CrI)^b^ | | | | |  |
|  | Human stool (n=211) | Chicken (n=136) | Duck (n=75) | Pig  (n=65) | Rodent (n=37) |  |  | Human blood (n=148) | Chicken (n=136) | Duck (n=75) | Pig  (n=65) | Rodent (n=37) |  |
| **ST** | 55.7 (41.3-67.6) | 38.7  (26.4-51.0) | 2.6  (0.2-7.7) | 1.4  (0.1-6.7) | 0.7  (0.0-3.8) |  |  | 26.7  (16.9-36.1) | 19.7  (10.9-28.6) | 2.1  (0.1-7.3) | 25.6  (16.4-34.8) | 25.1  (18.3-32.8) |  |
| **AMR** | 17  (4.9-29.8) | 36.5  (23.9-48.5) | 12.2  (2.3-26.2) | 30.3  (16.9-46.1) | 2.8  (0.1-11) |  |  | 7.8  (1.7-16.4) | 7.3  (0.4-20.7) | 24.0  (11.8-39.9) | 45.2  (30.5-58.7) | 13.5  (2.2-27.4) |  |
| **ST-AMR** | 31.8  (18.6-46.4) | 53.5  (39.9-66.1) | 3.8  (0.7-9.6) | 7.4  (2.3-16.0) | 1.9  (0.2-6.3) |  |  | 11.0  (5.8-18.2) | 10.8  (4.4-19.3) | 7.5  (3.2-14.0) | 40.2  (31.7-49.4) | 29.6  (22.0-37.4) |  |
|  |  |  |  |  |  |  |  |  |  |  |  |  |  |
| **2. Model with unobserved sources** | | | | | | | | | | | | | |
|  | **Human blood (n=148)** | | | | | |  | **Human stool (n=211)** | | | | | |
|  | Mixture coefficient α (x 100)^a^  (95% CrI)^b^ | | | | | |  | Mixture coefficient α (x 100)^a^  (95% CrI)^b^ | | | | | |
|  | Human stool (n=211) | Chicken (n=136) | Duck (n=75) | Pig  (n=65) | Rodent (n=37) | Unobserved source |  | Human blood (n=148) | Chicken (n=136) | Duck (n=75) | Pig  (n=65) | Rodent (n=37) | Unobserved source |
| **ST** | 44.1  (31.8-59.7) | 36.0  (20.9-48.2) | 2.2  (0.1-7.3) | 1.8  (0.0-7.0) | 0.7  (0.0-3.5) | 12.8  (7.7-24.8) |  | 2.8  (0.2-11.3) | 34.4  (20.5-47) | 2.5  (0.2-8.5) | 2.0  (0.1-8.4) | 1.2  (0.0-4.9) | 54.7  (42.7-67.3) |
| **AMR** | 6.2  (0.3-25.2) | 19.4  (3.0-37.6) | 4.9  (0.1-20.1) | 31.2  (13.4-46.5) | 12.3  (0.6-26.8) | 21.9  (14.6-31.0) |  | 2.3  (0.1-10.3) | 5.7  (0.3-18.5) | 21.7  (8.0-38.2) | 45.3  (32.1-58.5) | 16.8  (3.1-30.6) | 5.4 (2.4-9.7) |
| **ST-AMR** | 2.8  (0.2-11.3) | 34.4  (20.5-47) | 2.5  (0.2-8.5) | 2.0  (0.1-8.4) | 1.2  (0.0-4.9) | 54.7  (42.7-67.3) |  | 2.6  (0.3-7.6) | 6.2  (0.6-15.2) | 2.4  (0.1-8.1) | 28.4  (20.9-35.8) | 6.1  (0.7-15.7) | 52.9  (44.8-61.4) |

^a^Expressed as percentage

^b^95% credibility interval

**
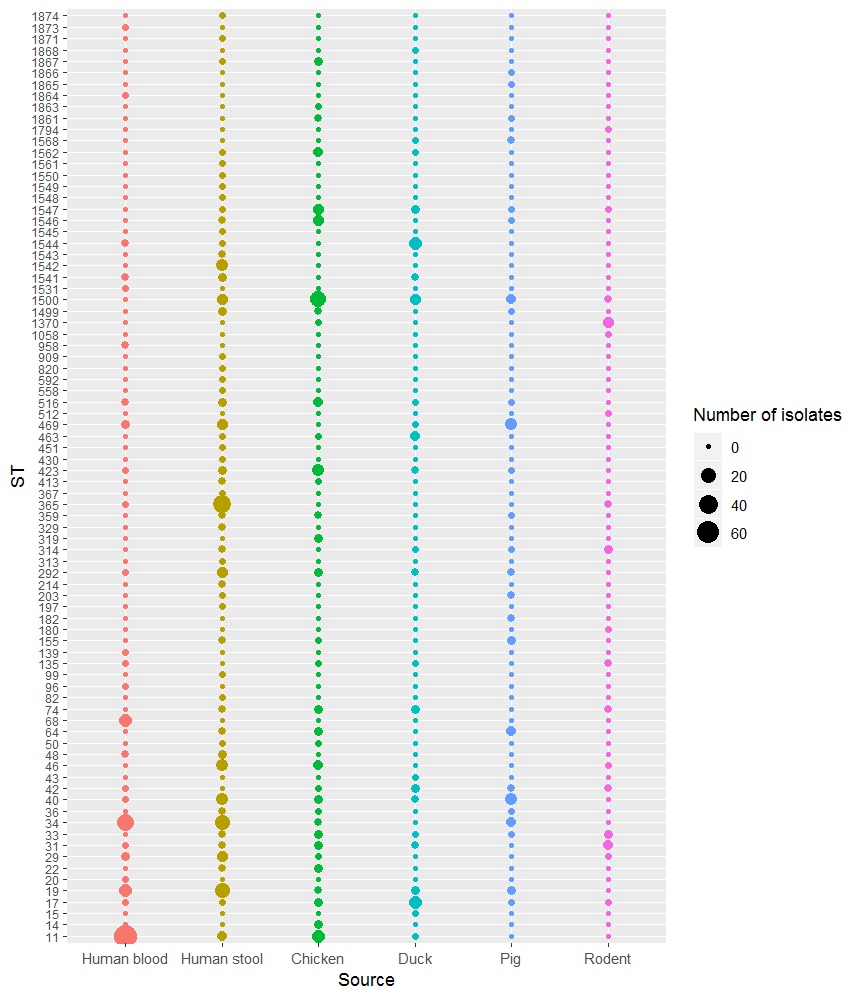
**

**Figure S1. Distribution of sequence types by the source of isolate**

**
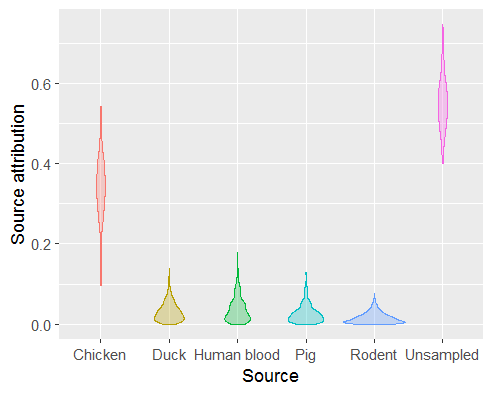

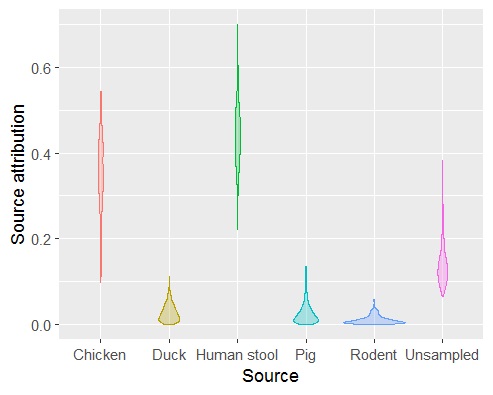
A. B.**

**Figure S2. Source attribution of human NTS isolates in Vietnam by sequence type.**

**
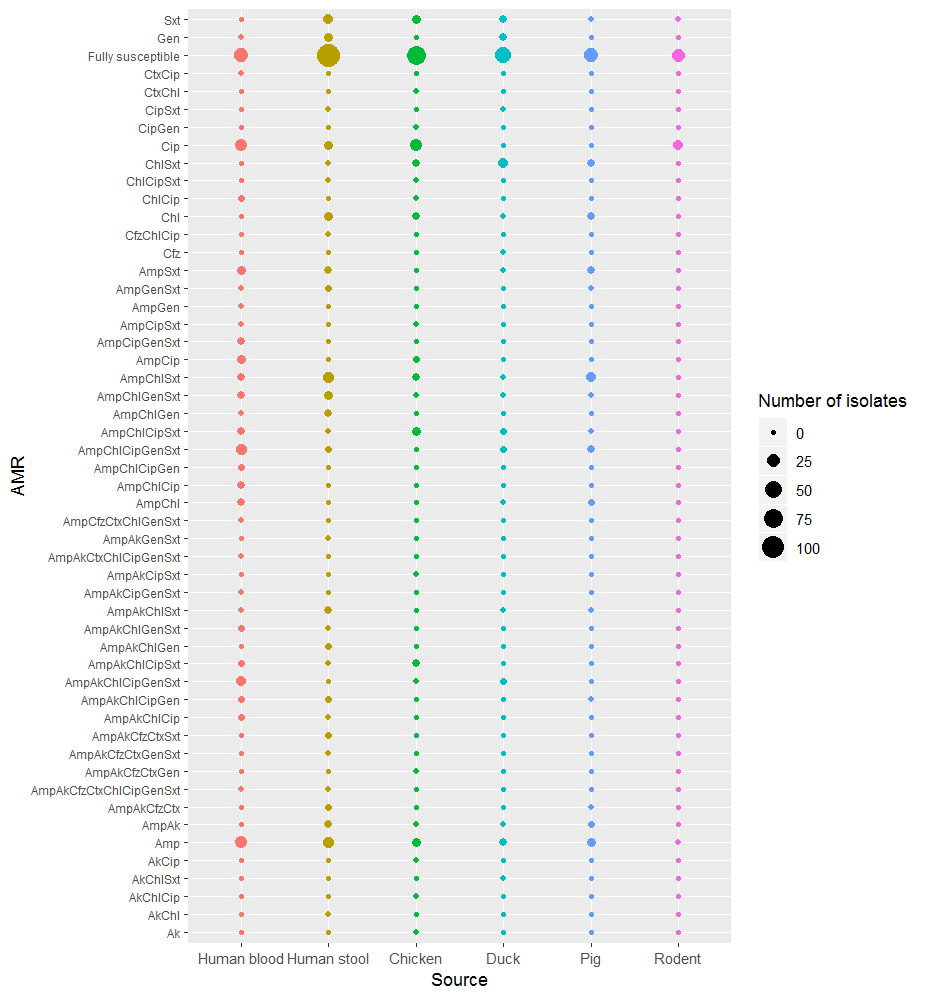
**

**Figure S3. Distribution of AMR profiles by the source of isolate.**


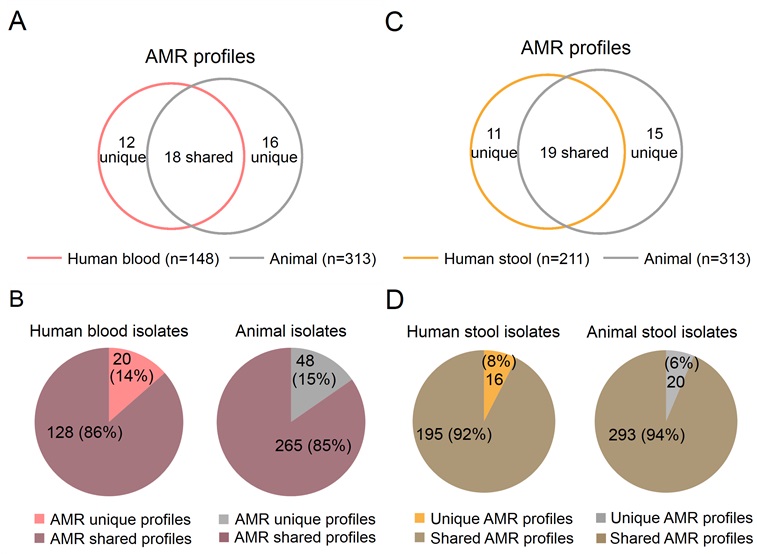


**Figure S4. Number and proportion of unique and shared AMR profiles among human and animal NTS isolates in Vietnam.**

**
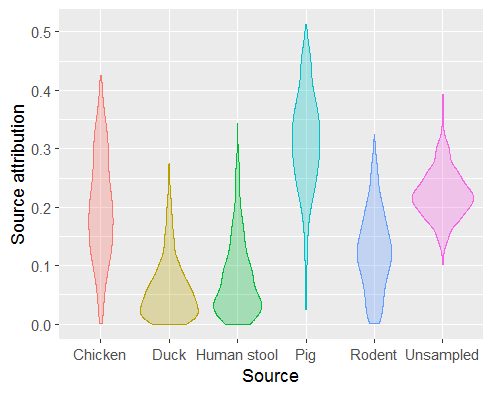

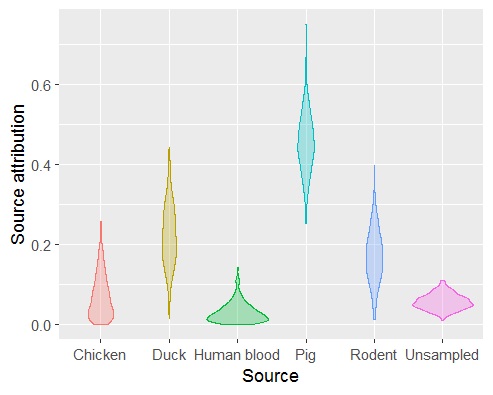
A B**

**Figure S5. Source attribution of NTS isolates in humans by AMR profile in Vietnam.**

**
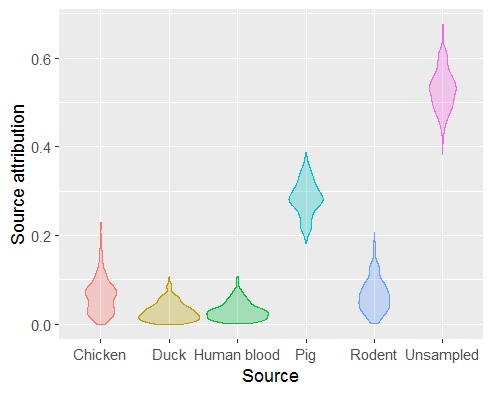

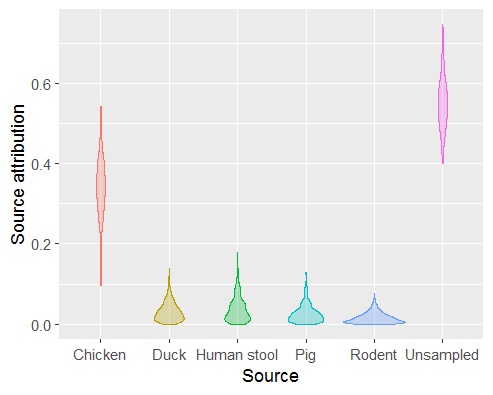
A B**

**Figure S6. Source attribution of NTS isolates in humans by ST-AMR profile in Vietnam.**
